# A practical contemporary approach to decision-making on subclinical hypothyroidism

**DOI:** 10.20945/2359-3997000000317

**Published:** 2020-12-15

**Authors:** José Augusto Sgarbi, Laura Sterian Ward

**Affiliations:** 1 Faculdade de Medicina de Marília Divisão de Endocrinologia e Metabolismo Unidade de Tireoide Marília SP Brasil Unidade de Tireoide, Divisão de Endocrinologia e Metabolismo, Faculdade de Medicina de Marília (Famema), Marília, SP, Brasil; 2 Universidade Estadual de Campinas Faculdade de Ciências Médicas Laboratório de Genética Molecular do Câncer Campinas SP Brasil Laboratório de Genética Molecular do Câncer, Faculdade de Ciências Médicas (FCM), Universidade Estadual de Campinas (Unicamp), Campinas, SP, Brasil

**Keywords:** Subclinical hypothyroidism, thyrotropin, levothyroxine, update, treatment

## Abstract

Subclinical hypothyroidism (Shypo) is an increasingly frequent condition in common medical practice. Its diagnosis continues to pose a challenge since a series of non-thyroidal and temporary conditions can elevate serum TSH levels. In addition, the consequences of Shypo are still up for debate. Although detrimental cardiovascular effects have been consistently demonstrated in the young, they are less evident in older adults (65-79 years), and even more so in the oldest old (≥80 years). In the absence of evidence of any benefits of treating Shypo in patients’ clinical manifestations and unfavorable outcomes, the most effective decision-making approach should include a thorough investigation of the patient's condition integrating all relevant clinical data, such as TSH levels, age, quality of life, comorbidities, cardiovascular risk, safety, and personal preferences. The decision-making process needs to take into account the risk of levothyroxine overtreatment and the resulting adverse consequences, such as reduction of bone mineral density, heart failure, and atrial fibrillation. Hence, current evidence suggests that individuals with TSH > 10 mU/L, who test positive for TPO Ab or are symptomatic may benefit from levothyroxine treatment. However, a more cautious and conservative approach is required in older (≥65 years of age), and oldest-old (≥80 years) patients, particularly those with frailty, in which the risk of treatment can outweigh potential benefits. The latter may benefit from a wait-and-see approach.

## INTRODUCTION

Management of subclinical hypothyroidism (Shypo), a condition biochemically defined by serum elevated thyroid-stimulating hormone (TSH) levels in the face of normal concentrations of free T_4_ (fT4), is one of the main challenges in current thyroid clinical practice (1–3). Shypo is the most frequent thyroid dysfunction in the general population, affecting up to 10% of iodine-sufficient populations, and is more prevalent in women, in whites, and in elderly people (4). In North America (5), the prevalence of Shypo has been estimated between 4.3% and 9.5%; in Europe, it is around 6.7% (6); and in Latin America, Brazil has been considered to be the country with the greatest prevalence of Shypo (4), varying from 5.4% (7) to 9.0% (8).

Caution needs to be taken in the diagnostic and therapeutic management of patients. Of note, non-thyroidal causes of Shypo and transient elevation of TSH levels must be excluded before considering treating a patient (9–12). Over the last decades, a growing body of evidence has associated Shypo with dyslipidemia (13–15), coronary heart disease (CHD) (16,17), stroke (18), and heart failure (HF) (19–21). However, despite advances in the knowledge of the clinical significance of Shypo, many uncertainties remain in practical management: it is usually an asymptomatic and frequently undiagnosed condition in the general population, and whether to screen individuals is still debated (22). Young and old are affected differently (23). Diagnostic criteria and therapeutic management of elderly patients are not clear (24). Moreover, no powered randomized clinical trial (RCT) has shown any clinical benefits from levothyroxine therapy, which remains controversial (22–28). Consequently, a series of factors need to be considered before deciding to treat or not a patient, including all the available evidence, clinical data, safety concerns, and patient preferences (29).

In this review, we summarize up-to-date information on the current evidence that can underpin the clinical management of patients with Shypo in adults (>18 years of age), except pregnant women. We focused on Medline-PubMed English literature and Cochrane Library databases using the term “subclinical hypothyroidism” combined with “differential diagnosis”, “natural history”, “quality of life”, cognitive symptoms”, “depression”, “dyslipidemia”, “cardiovascular disease”, “heart failure”, “stroke”, “mortality”, “age”, “treatment”, and “levothyroxine”. We prioritized systematic reviews, meta-analyses, randomized-controlled clinical trials, and high-quality prospective observational studies. Guidelines and relevant reviews were also included.

## DIAGNOSIS

Determination of serum TSH levels should be performed if hypothyroidism is suspected or as a case-finding strategy for specific high-risk groups of patients, such as women over 35 years of age, patients with a previous personal or family history of thyroid disease, type 1 diabetes, or other autoimmune diseases, Down and Turner syndromes, dyslipidemia, depression, or in patients using lithium or amiodarone (9–11). In addition, the Latin American Thyroid Society has proposed including both metabolic syndrome and type 2 diabetes patients among high-risk groups for hypothyroidism (30).

The diagnosis of Shypo can be confirmed by the presence of high serum TSH levels and normal fT4 concentrations (9–12). However, Shypo must be differentiated from other non-thyroidal causes of elevated serum TSH levels and normal fT4 concentrations, summarized in Table 1. Special attention should be paid to elevated TSH levels in the elderly, severely obese, critically ill, or hospitalized patients (3). The normal upper limit for TSH values may physiologically increase in healthy elderly people as a consequence of a reduced metabolic status or as an adaptive mechanism of protection against the catabolism of the aging process (24). Therefore, it is essential to differentiate the physiological increase of TSH levels from real thyroid dysfunction, bearing in mind that the diagnosis of Shypo should take into account the normal TSH reference ranges for the age (31). A Brazilian study suggested that TSH upper levels correspond to 4.3 mU/L for individuals between 20 and 59 years, 5.8 mU/L for those between 60 and 79 years, and 6.7 mU/L for those over 80 (32). It has been demonstrated that the prevalence of Shypo in patients with obesity is relatively high (33), but the diagnosis of Shypo may be complex in the severely obese, particularly with body mass index ≥ 40 kg/m^2^ (3), since a slight increase in TSH levels (usually < 8 mU/L) can occur as a consequence of the direct stimulatory effect of leptin on the hypothalamic neurons secreting the thyrotropin stimulating hormone (34,35), which is not a true thyroid disease. Thus, in the case of a severely obese patient, the presence of positive TPOAb and TSH levels ≥ 10 mU/L suggests Shypo. Finally, in critically ill, hospitalized patients, alterations in thyroid function tests can be found without the presence of any thyroid disease (36). Serum TSH levels may increase during the recovery phase of acute illnesses, although levels usually do not exceed 20 mU/L (9,12). Therefore, under such circumstances, denominated “euthyroid sick syndrome” or “non-thyroidal illness syndrome” (36), investigation of thyroid dysfunction should be avoided or requested only if there is strong suspicion of thyroid gland dysfunction (9,12). Other less common non-thyroidal causes (Table 1) of increased serum TSH levels include artifactual assay interferences by heterophilic antibodies or macro TSH, untreated Addison's disease, and thyrotropin resistance by mutation of the TSH-receptor (1–3). Transient causes of raised TSH, listed in Table 1, should also be excluded before considering treatment. Consistently, available guidelines (9–12) have recommended repeating measurements within 3-6 months to exclude laboratory errors or temporary causes of TSH elevation. Only patients with persistent Shypo should be considered for treatment.

**Table 1:** Causes of elevated TSH and normal fT4 concentrations

Thyroidal causes
Autoimmune thyroiditis (silent, post-partum and Hashimoto disease) Subacute and other forms of thyroiditis Thyroid lobectomy Post radioiodine therapy to treat Graves’ disease Post external radiotherapy of the cervical region Inadequate treatment of overt hypothyroidism Drug-induced (amiodarone, lithium, interferon-alpha, tyrosine-kinase inhibitors, immune checkpoint inhibitors)
Non-thyroidal causes
Physiological increase in elderly people Severe obesity Recovery from non-thyroidal illness Untreated Addison's disease Assay interferences (heterophilic antibodies, macro TSH) Thyrotropin resistance (mutation in the TSH-receptor)
Transient causes
Autoimmune thyroiditis (silent and post-partum thyroiditis) Subacute and other forms of thyroiditis Recovery from non-thyroidal illness Discontinuation of chronic levothyroxine treatment in euthyroid patients Post radioiodine therapy to treat Graves’ disease Drug-induced

## Clinical significance

Shypo has been categorized into two categories of severity: mild-to-moderate or grade 1 Shypo, when the serum TSH level is between 4.5 and 9.9 mU/L, and more severe or grade 2 Shypo, when serum TSH levels are ≥ 10 mU/L. Grade 2 Shypo patients are more likely to progress to overt hypothyroidism (OH), show symptoms, and develop unfavorable long-term results, and therefore are likely to have more benefits from treatment than those with grade 1 Shypo (3,12).

### Risks of progression to overt hypothyroidism

It is not completely understood why some patients with Shypo progress to OH, whereas others remain in Shypo or spontaneously regress to euthyroidism. Some individual or populational characteristics appear to influence the natural course of Shypo, such as the iodine level of the population, sex, age, and initial TSH levels (4). There are few population-based studies that evaluated the natural course of Shypo, none in Brazil. In the Whickham cohort (37), the annual progression rate to OH was greater in individuals with serum TSH levels > 6.0 mU/L and in the presence of anti-thyroid peroxidase antibody (TPOAb). In a large community study (38), 62% of the participants had normalized their elevated serum TSH levels in a second determination. Higher TSH levels and positive TPOAb were independently associated with a lower likelihood of reversion to euthyroidism in the elderly, while TSH ≥ 10 mU/L was independently associated with progression to OH (39). Prospective studies (40,41) also indicated that the rate of OH incidence was significantly greater among patients with TSH levels ≥ 10 mU/L and positive TPOAb. A Brazilian study (42) found that patients with positive thyroid ultrasound aspects of autoimmune thyroiditis were more likely to progress to OH. In conclusion, patients with TSH levels ≥ 10 mU/L and with thyroid autoimmunity have a greater risk of progression to OH.

### Quality of life, cognitive function, and depression

Shypo is associated with no or few clinical manifestations of hypothyroidism, usually unspecific symptoms (1–3). A large population-based study reported significant hypothyroidism-related symptoms in individuals with Shypo compared to those in euthyroidism (43), but other studies have failed to find similar results (44,45). Although a meta-analysis (46) demonstrated a significant relationship between Shypo and cognitive impairment in participants under 75 years of age (but not in those aged 75 or over), another meta-analysis (47) did not find any association. Data on the association of Shypo and depression are also conflicting (48–50). In the elderly, no clear association between Shypo and worsening quality of life, cognitive decline, or depression has been consistently reported (51–57). There are scant RCTs on the benefit of levothyroxine therapy in patients with Shypo. Levothyroxine therapy was associated with a significant improvement of symptoms of tiredness, muscle strength, and quality of life in two small studies (58,59), but another RCT found no relevant benefit to health-related quality of life from 6 months of levothyroxine therapy in women with Shypo (60). Of note, in a recent meta-analysis of 21 studies including 2,192 adults, levothyroxine therapy provided no improvement in quality of life or thyroid-related symptoms (61). Moreover, RCTs have been unable to demonstrate any apparent benefit of treating both older (≥65 years) and oldest-old (≥80 years) patients with Shypo. (62–64). Thus, there is no clear evidence that the treatment of Shypo is associated with improved quality of life, cognitive function, or depression.

### Dyslipidemia

Thyroid hormones have a marked effect on lipid metabolism, including upregulation of LDL-c receptors and inhibition of LDL-c oxidation (15). Shypo has been associated with increased total cholesterol and LDL-c in small and population-based studies (14), and findings derived from randomized double-blind studies (58,64–66) and meta-analysis (13,67) have shown favorable effects of Shypo treatment on lipid profile. Besides, recent meta-analyses have reported an association between Shypo and endothelial dysfunction (68) with beneficial effects from levothyroxine therapy (69,70). Shypo has also been associated with a higher risk of metabolic syndrome, although controversies persist regarding this association (71).

### Cardiovascular risk and mortality

The thyroid hormone regulates the structure and function of the heart through genomic and non-genomic actions with an influence on cardiac growth, myocardial activity, and vascular function (72). A set of heart abnormalities has been associated with both OH and Shypo, including altered ventricular contractility and relaxation dynamic, compromised cardiac function, and heart failure (HF) (73,74). A mild thyroid hormone deficiency may reduce the entry of calcium into the myocyte, increase the transcription of β-myosin and decrease that of α-myosin, leading to reduced myocyte cell contractile capacity and cardiac atrophy (19,74). This mechanism could partially explain the significant association between Shypo and a greater risk of HF incidence, particularly in patients with TSH ≥ 10 mU/L (20) and in the elderly (21). Moreover, Shypo appears to affect the prognosis of patients with HF, increasing the odds of hospitalization and death (75).

The most consistent data on the association of Shypo with cardiovascular risk comes from a meta-analysis (16) including individual data from more than 55,000 participants from 11 prospective studies. The risk of non-fatal CHD events was almost twice as high in participants with grade 2 Shypo compared to those in euthyroidism. Interestingly, the risk of fatal CHD events was about 1.5 times higher in both grade 1 and 2 Shypo. A more recent meta-analysis confirmed these results, especially for younger participants (≤65 years of age) and individuals at high cardiovascular risk (17). Another meta-analysis of prospective studies associated Shypo with a higher risk of fatal and non-fatal stroke events, but only in younger participants (18). However, the effects of levothyroxine treatment on cardiovascular risk remain doubtful. A recent double-blind randomized controlled study of 95 participants (mean age, 64 years) with Shypo and acute myocardial infarction failed to find any significant improvement in the left ventricular ejection fraction of patients treated with levothyroxine for 52 weeks (76).

Of note, the detrimental cardiovascular effects of Shypo appear to be well established in younger adults but are less evident in older adults (65-79 years), and even more so in the oldest old (≥80 years). In a 4-year follow-up of octogenarian participants, those with elevated serum TSH levels (up to 10 mU/L) were more likely to survive than those with lower TSH values (51). A meta-analysis including 2,531 Shypo participants from 15 studies showed that CHD incidence and cardiovascular/all-cause mortality rates were elevated in those aged 65 and younger, but not in older people (23). Furthermore, a retrospective analysis showed that Shypo patients under 70 treated with levothyroxine faced lower cardiovascular risk than untreated patients, but no benefit was found for those above 70 (77). Thus, Shypo appears to affect young and old people differently. In the elderly, a mild elevation of TSH (<10 mU/L) may reflect a normal age-related increase rather than a thyroid dysfunction (24,78).

## TREATMENT

Although Shypo has been consistently associated with increased cardiovascular risk and mortality, there is lack of evidence on the possible beneficial effect of levothyroxine replacement therapy on these risks (1–3,27). Thus, the decision of whether to treat a patient remains a challenge for clinicians and endocrinologists. Importantly, a recent meta-analysis including 2,192 participants from 21 randomized studies found no benefits in the quality of life or thyroid-related symptoms of patients with Shypo treated with levothyroxine (61). These findings resulted in a strong recommendation against thyroid hormone therapy in adults with Shypo, except for patients with TSH > 20 mU/L, with severe symptoms or those under 30 (27). However, this guideline has been contested (28,29). The majority of participants included in the meta-analysis (61) had grade 1 Shypo with mean baseline TSH values ranging from 6.4 to 7.3 mU/L, and at least one-third of them (n = 737) were derived from an RCT study (63) that included elderly patients ≥65 years, in which levothyroxine treatment provided no apparent benefits. Hence, it is not possible to generalize such results to higher-risk patients with grade 2 Shypo, since grade 1 Shypo, particularly in elderly patients, has been not consistently associated with significant clinical consequences (1–3).

In the absence of evidence of any benefits of treating Shypo in patients’ clinical manifestations and unfavorable outcomes, the most effective decision-making approach should include a thorough investigation of the patient's condition integrating all relevant clinical data, such as TSH levels, age, quality of life, comorbidities, cardiovascular risk, safety, and personal preferences (29). The approach starts with a precise diagnosis in which only patients without non-thyroidal causes of TSH elevation (Table 1) and persistent Shypo should be considered for treatment. The patient's age and the severity of Shypo according to TSH values are the main elements that guide the decision-making process.

### Younger patients (<65 years of age)

**Figure 1:**
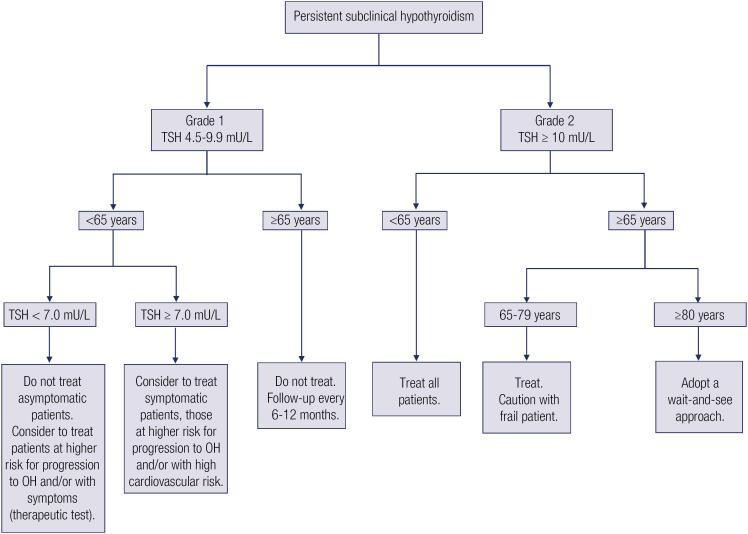
Algorithm for levothyroxine replacement therapy for patients with persistent subclinical hypothyroidism

The treatment has been recommended for all younger patients with grade 2 Shypo (TSH ≥ 10 mU/L) aiming to reduce the risk of progression to OH and of long-term cardiovascular complications and mortality (Figure 1). Conversely, no treatment is necessary, in general, for healthy and asymptomatic patients with grade 1 Shypo (TSH 4.5-9.9 mU/L) (9–12). However, treatment may be considered for patients with grade 1 Shypo if serum TSH ≥ 7.0 mU/L, particularly for those with pre-existing cardiovascular disease or high cardiovascular risk, due to the association with a higher risk of fatal and non-fatal CHD and stroke in this context (3,10,12,16). Clinicians can also consider treating patients with grade 1 Shypo at higher risk of progression to OH (female gender, a progressive increase of TSH levels, positive TPOAb, or US pattern of autoimmune thyroiditis). Moreover, a therapeutic test with levothyroxine for a determined period (at least three months) may be performed in patients with hypothyroidism-related symptoms. If no symptom relief is observed after TSH normalization, treatment should be stopped (Figure 1) (9–12).

### Older patients (≥65 years of age)

The clinical approach to elderly patients with Shypo requires even more caution. On the one hand, these patients are less likely to progress to OH (39,78) and there is no clear evidence of the association of Shypo with worse quality of life, symptoms related to hypothyroidism, cognitive impairment, and depression (51–57), or with unfavorable cardiovascular outcomes (23,24,51,77). Furthermore, no RCT study (62) or meta-analysis (60) has demonstrated a clear benefit of treatment in this subgroup of patients in terms of thyroid-related symptoms, quality of life, and cognition function. Finally, large studies failed to show any significant beneficial effect of levothyroxine therapy in older people > 65-79 years regarding fatal- and nonfatal cardiovascular events and all-cause mortality (23,77–80).

On the other hand, older people are at higher risk of levothyroxine overtreatment and are more susceptible to significant adverse consequences, such as reduction of bone mineral density (81), heart failure (21), and atrial fibrillation (82,83). Of note, a Brazilian multicenter study including 2,292 patients with hypothyroidism found that 14.4% were using supraphysiological doses of levothyroxine (84). Together, these data reinforce the importance of a decision-making process based on personal contexts and a careful balance between risks and benefits. Guidelines (9–12) have recommended the treatment of Shypo in older patients only when TSH ≥ 10 mU/L, particularly if at a higher cardiovascular risk (Figure 1). However, a wait-and-see approach has been proposed for the oldest old (≥80 years of age) (1,3,12,24) and frail patients (78), aiming to avoid levothyroxine therapy because these patients are at higher risk of harmful effects and overtreatment (1,12,24,77,78). Levothyroxine replacement therapy should be considered for this set of patients only when TSH levels are above 10 mU/L and there are clear symptoms of hypothyroidism and/or high cardiovascular risk (1,12,24,78).

### How to treat

The treatment of Shypo must follow the same principles as the treatment of OH. In summary, the drug of choice is levothyroxine, and there is no recommendation for combined therapy T4 + T3, particularly in elderly or frail patients. The initial dose varies depending on the patient's context, such as the severity of Shypo, age, and presence of comorbidities, usually no more than 50 µg/day. Starting doses of 25 µg/day and 12 µg/day should be recommended for older (65-79 years) and oldest-old (≥80 years) patients, respectively, with progressive titration every 4-8 weeks considering optimal TSH target values from 2,5 to 6 mU/L (1–3,9,24,78).

In conclusion, Shypo is highly prevalent in the general population and increasingly common in medical practice; if not recognized and treated, it can be associated with long-term important complications. However, a screening strategy is still controversial because of the lack of consistent evidence on the benefits of treatment. Therefore, the decision to treat should rely on the available evidence concerning the risks of not treating, on the patient's characteristics, and on individual clinical judgment. A careful individualized approach aiming to identify patients with persistent Shypo who could benefit from levothyroxine therapy is mandatory. Patients under 65 with grade 2 Shypo (TSH ≥ 10 mU/L) or with grade 1 Shypo (TSH > 7.0 mU/L) in the presence of pre-existing high cardiovascular risk should be treated aiming to reduce long-term cardiovascular complications. Treatment can also be considered in younger patients and grade 1 Shypo (4.5-9.9 mU/L) at a high risk of progression to OH or in patients who are symptomatic. However, a more cautious and conservative approach is required in older (65-79 years of age), and oldest-old (≥80 years) patients, particularly those with frailty, in which the risk of treatment can outweigh potential benefits.
